# Computer and laboratory simulation in the teaching of neonatal nursing: innovation and impact on learning**^1^**

**DOI:** 10.1590/1518-8345.1005.2808

**Published:** 2016-10-10

**Authors:** Luciana Mara Monti Fonseca, Natália Del' Angelo Aredes, Ananda Maria Fernandes, Luís Manuel da Cunha Batalha, Jorge Manuel Amado Apóstolo, José Carlos Amado Martins, Manuel Alves Rodrigues

**Affiliations:** 2 PhD, Professor Associado, Escola de Enfermagem de Ribeirão Preto, Universidade de São Paulo, WHO Collaborating Centre for Nursing Research Development, Brazil; 3 MSc, Doutoranda, Escola de Enfermagem de Ribeirão Preto, Universidade de São Paulo, Centro Colaborador da OMS para o Desenvolvimento da Pesquisa em Enfermagem, Brazil; 4 PhD, Professor, Escola Superior de Enfermagem de Coimbra, Coimbra, Portugal; 5 PhD, Adjunct Professor, Escola Superior de Enfermagem de Coimbra, Coimbra, Portugal; 6 PhD, Full Professor, Escola Superior de Enfermagem de Coimbra, Coimbra, Portugal

**Keywords:** Neonatal Nursing, Educational Technology, Learning, Physical Examination, Simulation

## Abstract

**Objectives:**

to evaluate the cognitive learning of nursing students in neonatal clinical evaluation from a blended course with the use of computer and laboratory simulation; to compare the cognitive learning of students in a control and experimental group testing the laboratory simulation; and to assess the extracurricular blended course offered on the clinical assessment of preterm infants, according to the students.

**Method:**

a quasi-experimental study with 14 Portuguese students, containing pretest, midterm test and post-test. The technologies offered in the course were serious game e-Baby, instructional software of semiology and semiotechnique, and laboratory simulation. Data collection tools developed for this study were used for the course evaluation and characterization of the students. Nonparametric statistics were used: Mann-Whitney and Wilcoxon.

**Results:**

the use of validated digital technologies and laboratory simulation demonstrated a statistically significant difference (p = 0.001) in the learning of the participants. The course was evaluated as very satisfactory for them. The laboratory simulation alone did not represent a significant difference in the learning.

**Conclusions:**

the cognitive learning of participants increased significantly. The use of technology can be partly responsible for the course success, showing it to be an important teaching tool for innovation and motivation of learning in healthcare.

## Introduction 

Among the strategies and tools applied to nursing education, realistic simulations conducted in laboratories, navigation software organized into specific content, and *serious games* (educational games) are important.

*Serious games* are characterized as a complementary tool, whose main purpose is to provide experience and emotion by means of a simulated virtual environment transformed into meaningful learning^1^ providing support for education with solid concepts based on critical thinking, problem solving, planning, flexibility and adaptability^2^.

In addition to the digital tools for education in healthcare, the simulated practice used in education, presents advantages indicated in the literature, with an emphasis on: *Patient safety*, as the student will have his first practical experience with a real patient; *Ethics* in the care performed by students in the healthcare area, because they will be better prepared for real situations after the previous simulation and *Learning opportunity,* considering that not all health intervention situations can be performed by students - such as emergency situations, for example ^(^ ^3^ ^-^ ^4^.

This study was developed to understand the impact of a *serious game*, with computer simulation, associated with the laboratory simulation in a blended course on clinical evaluation of preterm infant.

## Objectives

The objectives of this study were: to evaluate the cognitive learning of nursing students regarding neonatal clinical assessment from a blended course using computer and laboratory simulation; to compare the cognitive learning of the students in the control and experimental group when testing the laboratory simulation; and to evaluate the extracurricular blended courses offered on clinical assessment of preterm infants, according to students.

## Methods

This was a quasi-experimental research study with 14 Portuguese students whose intervention was composed of the stages of the *Clinical assessment of preterm infant* course, which included an active pedagogical strategy, understood in this study as educational activities in which the student is encouraged to be an active member in the teaching-learning process, by means of challenges and questions.

Interactive tools were used, such as the s*erious game e-baby* ^5^ (which virtually simulates the clinical assessment of a preterm infant with respiratory problems maintained in the incubator), instructional *software* semiology and semiotechnique of the preterm newborn (SSRNPT)^6^, and laboratory simulation.

### Data collection

The blended course was offered in an extracurricular certificate program, by the School of Nursing of Coimbra (ESEnfC), Portugal. It was available in the Moodle virtual learning environment, with total time of 30 hours (15 days), administered in the steps of pre-test, midterm test, and post-test of knowledge.

Student knowledge (pre-test) was verified on the first day of the course and the inaugural class attendance on prematurity and the importance of clinical assessment for caring for this customer. After class attendance, other planned activities for the week were developed, aiming to facilitate the recognition of the *e-Baby* and SSRNPT digital technologies.

A second physical meeting occurred after the presentation of digital tools to the students, at the end of the first week of the course, at which point the midterm test and simulation laboratory were performed, focused on the nursing care for a premature infant with a health impairment requiring oxygenation.

**Figure 1:**
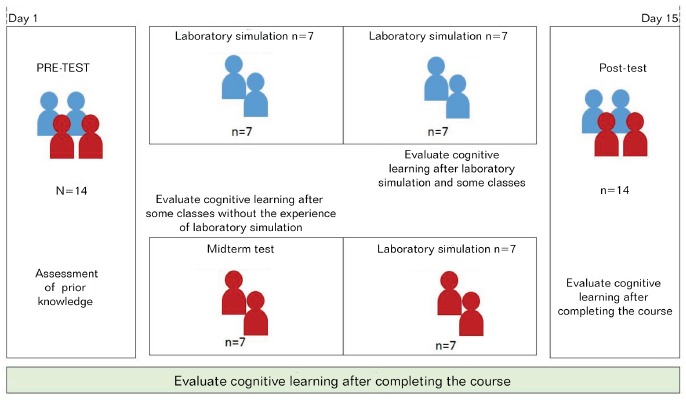
Implementation of the study methodology

For analysis of the laboratory simulation as intervention research, and so that the proposed methodological model could be used, all students in the sample (n=14) separately participated in this laboratory activity, considering the aim of this study (Figure 1), immediately before the midterm test (experimental group n=7/50%) and after the midterm test (control group n =7/50%).

Thus, Figure 1 represents the study flowchart for a better understanding of the method applied and the chronology of the study procedures.

The second week of the course, after the laboratory simulation, was intended for the use of online digital technologies and a theme-related discussion, by means of *Moodle* activities: discussion forum, chat and e-mail. The last physical meeting occurred in the 15th day of course. In that meeting, the post-test for final evaluation of student learning, course evaluation and certification of participants by the educational institution were conducted.

Three online tools available on Moodle was used, one for characterization of the subject, one for the tests of cognitive learning (pre-test, midterm, and post-test) and one for the course evaluation.

The characterization included issues related to personal data, such as sex and age, as well as to identify the technologies used routinely by the students.

The learning performance was evaluated by means of a questionnaire composed of 20 objective questions, exclusively designed for this study (with multimedia), used for all the tests, but in a randomized order of appearance of the questions and its alternatives, ensuring the same level of difficulty. The questions were designed to measure cognitive learning (conceptual knowledge hierarchically organized according to the degree of abstraction and generalization, which were related to each other) ^7^.

For the test of learning, the range of scores was a minimum of 0 and a maximum of 100. The desirable score was greater than or equal to 70.

Performance, established as the difference between scores achieved by students in a subsequent test and a previous test, the, difference between the post-test and pretest scores, and from the midterm test and the pre-test score ^8^.

The instrument for the course evaluation by students was composed of 23 items using a Likert scale, which used the scoring of: "insufficient", "sufficient", "good", "very good" and "excellent." A free text space was included for expressions of their opinions on the course, enhancing or adding information regarding the instrument items. This instrument was developed for this course and included important aspects for the teachers to identify strengths and opportunities for improvement, according to the empirical experience of researchers, as well as teachers.

The items present in the instrument included the satisfaction and motivation of students for the course, the practical activity in the simulation center, the access to digital technologies, and the implementation of a forum and chat in the virtual environment. In addition, the items investigated the relevance of all the activities and teaching tools that supported the course, in the same way as the general evaluation for organization and relevance.

### Data analysis 

The general description of the data relating to the characterization of the students was presented by use of descriptive statistics (mean, standard deviation, median and semi-quartile range).

The statistical analysis tests were performed using the *Statistical Package for the Social Sciences* (SPSS), non-parametric analysis method, according the nature of the main variable of interest which was poorly understood, and the limited sample size.

The intra-group comparison was performed using the non-parametric Wilcoxon test, while the comparison between control and experimental group was analyzed using the Mann-Whitney test, both of which considered a significance level of p < 0.05 and 95% of confidence interval.

### Ethical aspects

This study was submitted to the Ethics Committee of the Research Unit in Health Sciences - Nursing, of the Nursing School of Coimbra, and approved under the protocol No 73-02/2012. The rights provided in the *informed consent forms* (ICF) were explained by the researcher, exempting the volunteer of any benefit or harm, according to the recommendations and standards of Good Clinical Practice. Because of the relationship of authority, the institution's teachers linked to the participants were not included in the invitation and presentation of the ICF, which was conducted by a non-teaching researcher of ESEnfC.

## Results

### Characteristics of the participants

Among the 14 (100%) students who participated in the study, 12 (85.7%) were women between 21 and 29 years of age (five of them with 21 years, another five with 22, three with 23, and one student with 29 years). None of the participants in this study reported working, but rather were exclusively dedicated to their academic pursuits. All of them owned a computer and had internet access. Regarding the frequency of computer use, 11 (78.6%) stated a frequent use, while others reported a regular use and, in relation to the principal place of computer access, nine (64.3%) reported at home, four (28.6%) at the university, and one (7.1%) at the home of relatives and friends. All (100%) reported having a computer and, 30% had a laptop (notebook) and 70% a desktop; all connected to the internet (100%).

All the students reported have already used the internet to support the activities of their university courses, and pointed as the main objectives of the network the sending and receiving of e-mails and conducting research (both reported for n = 8/57.2%), participation in social networks and leisure (both reported for n = 4/28.6%).

Among the participants, 12 (85.7%) never used educational technologies and two (14.3%) had used them previously. Half of our sample (n=7/50%) accessed internet games, citing those developed in Flash^(r)^, Facebook^(r)^ games, Puzzle Bubble^(r)^ with strategy of nature, reasoning, and other assorted entertainment.

All students (n = 14/100%) reported that this was their first participation in a course supported by educational technologies, but all of them had already participated in a simulation laboratory previously.

### Cognitive learning before and after the course

Data regarding scores obtained at the different periods of evaluation, according to measures of central tendency, are presented in Table 1. The total sample is used in order to verify the development of students' learning, regardless of the simulation occurring before or after the midterm test.

**Table 1:** Measures of central tendency of the scores obtained by students in different periods of evaluation. Coimbra, Portugal, 2012

Measures of central tendency		Pre-test score	Midterm test score	Poss-test score
n				
	Valid	14	14	14
	Loss	0	0	0
Mean		53.9	68.2	90.3
Median		60.0	70.0	95.0
Mode		40.0	70.0	90.0
Standard deviation		15.2	13.5	12.78
Minimum		20.0	40.0	55.0
Maximum		75.0	90.0	100.0
Percentile				
	25	40.0	60.0	90.0
	50	60.0	70.0	95.0
	75	65.0	77.5	100.0

A statistically significant difference (p=0.001) in the rate of correct answers on the issue addressed in the course was verified, regarding the cognitive learning of the students obtained by comparing the post-test and pre-test scores. This result refers to the whole sample, without division into groups; that is, this result indicates that, regardless of division of the groups, the learning in the subject improved significantly for all participants.

### Cognitive learning before and after the laboratory simulation (midterm)

Comparing scores obtained on the midterm test with the pre-test, no statistical significance was identified in the performance analysis of either group, expressed by a p-value (0.058 for the experimental and 0.062 for control) using the Wilcoxon test. During the course, specifically in the midterm testing phase, no statistically significant difference was demonstrated in the student learning of either groups. A statistical difference was identified only at the end of the course, as previously shown.

No statistically significant difference (p=0.845) in performance was obtained from the beginning until the midterm test, and p= 0.846 in the performance achieved from the beginning to the end of the course was identified by the Mann-Whitney test, when comparing the performance between the control and experimental groups. These data reveal that students from both groups had similar outcomes in results when comparing experimental versus control.

### Opinion of the students on the course 

Among the 14 participants, only one student did not respond to course evaluation instrument, alleging forgetfulness. The results were mostly positive, ranging between "very good" and "excellent" in all the variables evaluated. There was a negative assessment characterized by "insufficient" only in the item: " time reserved for simulation laboratory practice" by three students, - corresponding to 21.5% of the study sample.

In the space designed for the considerations, such as suggestions and criticisms, some comments emerged and are presented in three categories: laboratory simulation, educational technology, and the course in general. These comments were submitted by eight participants.

### Laboratory simulation 

*I think in the next course more time could be available for laboratory practice. And some more specific nursing intervention themes should be deepened, as some specific procedures in the preterm infant* (Student C);

*I think that the practices were very important and a whole day could be better for discussing other topics* (Student E);

*I think that a larger practical component would be necessary in the laboratory (at least in the evaluations of respiratory sounds)* (Student F);

*I suggest that there is more time and more diversity of practices should be available* (Student G);

*Maybe, the practical classes can be better exploited with more time available to observe the newborn, as a whole, and not focusing on the oxygenation* (Student L).

### Digital educational technology

*More situations in e-Baby could be introduced* (Student B).

### Course in general

*The course is very well organized. All information is very pertinent. The teacher availability, always available and interested in resolving all situations must be commended. The fact that e-learning allows students to develop their skills according to their level/learning time and always being able to return or stop the development and then perform according to the availability* (Student H).

## Discussion

Many studies show a significant increase in students' knowledge when using the simulation associated with other teaching strategies,^9^ and that the digital technology is a valuable resource in the acquisition of information. However, its use alone may not be reflected in the acquisition of knowledge^10^. This information is supported by the results of this study, in which a course based on active learning methodology and associated tools was offered (digital material, virtual learning environment including chats and forums, laboratory and virtual simulation). 

All educational intervention results improved learning, however, the relevance of the improvement verified by this study, based on the value of p=0.001 must be noted, despite the limitation of the sample size. A considerable improvement was identified, when analyzing the measures of central tendency, showing an increase of the mean score achieved by about 80% of the students (mean in the pre-test of 53.9 points and post-test of 90.3).

The goals of nursing higher education institutions are that the student can interpret and execute challenges, and search for creative solutions based on scientific theory. The decision making process must be developed by means of reflection about the problem to be solved and the use of critical thinking, associating the practice with theory. Researchers reinforce the need for critical analysis of the results of nurses' education, comparing this process with "praxis", in the context of the working process and the quality demands of the health system ^11^.

The improvement of the processes that includes the complex task of teaching and learning is the target of investigations throughout human history, studied since antiquity^12^. Increasingly, the teaching-learning process has been emphasized in the healthcare, due to the need to develop more creative professionals, engaged in problem-solving and guided by scientific evidence and ethics.

Studies attribute positive and successful characteristics to different strategies and tools used in this research, including: laboratory simulation, such as stimulating critical thinking^13^, virtual simulation as motivating and interactive with great potential as a pedagogical approach^14^, chats and forums as tools for online communication and collaboration^15^. 

The results showed that the laboratory simulation, when analyzed as an isolated intervention, did not represent a significant difference between the control and experimental groups. However, at the end of the course, and after the several activities, the students from both groups achieved a significant improvement in learning without difference in their performance. The association of different educational tools is advantageous and they should not be used singly^16^. In addition, one should make use of innovative and creative strategies for the teaching-learning process, according to some researchers who evaluated the relevance of teaching technologies articulated to active learning methods^17^.

A study^18^, conducted with 54 nursing students, analyzed clinical reasoning performance, comparing simulation by computer *versus* laboratory in simulation, in the active context of teaching-learning. It concluded that one strategy was not highlighted in relation to the other. This study provides a theoretical basis about the impact on learning, in which the laboratory simulation was not better than the other strategies used.

With regard to the participants' statements, the request for extension and expansion of educational opportunities and tools employed is frequent, corroborating the data obtained by means of the evaluation of satisfaction with the course. The enthusiasm of the participants with the simulations (laboratory and virtual) - emphasized in the statements - is fundamental because it is related to motivation and interest in the educational process. Therefore, studies that incorporate innovative tools very often assess user satisfaction as a way to measure positive or negative response, as evidenced by an integrative literature review ^(^ ^19^.

Despite satisfaction not necessarily being reflected in a good clinical performance^20^, it is a good indicator of quality education, as it is related to motivation and interest of students in learning, in addition to the versatility in simulating the clinical practice ^21^ ^-^ ^22^.

Considering that the E-Baby game virtually simulates the neonatal care, the satisfaction of students when integrated into the game in the educational context is perceived. Analyzing the positive impact of this strategy, the importance of promoting new challenges and introducing new teaching tools can be highlighted. 

The emotional design inculcated in the game development process may have reflected directly on the motivation to play E-Baby. Emotional design is an aspect of unique importance in the development of educational materials in the digital environment and in education; it consists of the search for alignment between the user's desire for using the educational tools and the feeling that you are emotionally integrated with them, translated in motivation and improvement of learning outcomes ^(^ ^23^. 

It is important that the teacher, when selecting the teaching tools that contribute to the teaching practice, recognizes their potentials and weaknesses, and understand their mechanisms, knowing how to use them. In other words, the teacher must identify the best way to use these tools in the activities offered to students.

The use of educational technologies has increased in recent years, and this is due to both the advancement of devices and programming languages, as well as to the preference of students and teachers. Especially, there is an interest among university students, called "digital natives", who have very different learning needs in relation to previous generations. Some researchers advocate that today's young people learn more easily when in contact with technology, with group tasks based on experiences and problems, and with collaborative and interactive learning ^24^.

However, it is necessary to evaluate the developed educational tools, so that the cost-benefit question with each innovation can be analyzed, with basis on their impact and service to the development proposal. In addition to the validations of computer-user interface and scientific content, there is the evaluation by the target population for evaluation of satisfaction as to the results obtained with its use ^(^ ^25^.

The teaching tools must be evaluated and improved by students and teachers, who are the end users. Thus, their use in education can be significant, again. The results presented in this study reflect the Portuguese students' satisfaction with the course, from its organization and composition through to the execution. We further suggest that the evaluation process of nursing courses is encouraged, includes self-evaluation, teacher evaluation by the students, and formative evaluation, just as was used by the researchers^26^, when analyzing and discussing the education of health professionals with a focus on problematization . Formative evaluation, among these, is a current challenge whose objective is monitoring the students' performance throughout the courses and not just at the end, allowing the educational reinforcement of the identified weaknesses.

## Conclusion

According to the results, the cognitive learning of participants increased significantly from the beginning until the end of the extracurricular course, with gradual increase in scores on the tests (pre-test, midterm test and post-test). We believe that the teaching tools used as a support for physical classes and online activities had a satisfactory impact on the results.

On the other hand, the laboratory simulation, when analyzed alone, did not significantly impact the students' test scores, which highlights the importance of the association of tools and strategies in nursing higher education.

Considering the evaluation of the simulations (virtual and laboratory), the students showed great satisfaction with participating and practicing in a safe and simulated fashion, in terms of decision making grounded in a situation that is common in a hospital neonatal unit scenario.

 The positive course evaluation indicates students' motivation and interest in learning, although the course was conducted during the vacation period of a Portuguese nursing school. *A priori*, the course completion in this period generated some anxiety, given the risk of students not to engage in the activities, but the results were much better than expected. There was an intense participation in the discussion and chat forums in the virtual environment, and access to digital technologies that could be used over the two weeks at any time and as the student wanted.

The small sample size is recognized as a limitation of the study, nonetheless it was possible to perform the statistical analysis with nonparametric tests. Further studies should be conducted investigating the impact on learning, with larger samples and technological innovation opportunities for education.

## References

[1] Marsh T (2011). Serious games continuum between games for purpose and experiential
environments for purpose. Entertainment Comput.

[2] Romero M, Usart M, Ott M (2015). Can serious games contribute to developing and sustaining 21st century
skills. Games Culture.

[3] Martins JCA, Mazzo A, Baptista RCN, Coutinho VRD, Godoy S, Mendes IAC (2012). The simulated clinical experience in nursing education a historical
review. Acta Paul Enferm.

[4] Gutierrez IH (2010). La simulación clínica como herramienta de evaluación de competencias
en la formación de enfermería. Reduca (Enfermería, Fisioterapia y Podologia)..

[5] Fonseca LMM, Dias DMV, Góes FSN, Seixas CA, Scochi CGS, Martins JCA (2014). Development of the e-baby serious game with regard to the evaluation
of oxygenation in preterm babies. Computers, Informatics, Nurs.

[6] Fonseca LMM, Góes FSN, Ferecini GM, Leite AM, Mello DF, Scochi CGS (2009). Inovação tecnológica no ensino da semiotécnica e semiologia em
enfermagem neonatal: do desenvolvimento à utilização de um software
educacional. Texto Contexto Enferm.

[7] Pelizzari A, Kriegl ML, Baron MP, Finck NTL, Dorocinski SI (2002). Teoria da aprendizagem significativa segundo Ausubel. Rev PEC..

[8] Domitrov DM, Rumrill PD (2003). Pretest-posttest designs and measurement of change. Work.

[9] Wilford A, Doyle TJ (2006). Integrating simulation training into the nursing
curriculum. Br J Nurs.

[10] Lerner C, Gaca AM, Frush DP, Hohenhaus S, Ancarana A, Seelinger TA (2009). Enhancing pediatric safety assessing and improving resident competency
in life-threatening events with a computer-based interactive resuscitation
tool. Pediatr Radiol.

[11] Corbellini VL, Santos BRL, Ojeda BS, Gerhart LM, Eidt OR, Stein SC (2010). Nexos e desafios na formação professional do
enfermeiro. REBEN.

[12] Padilha LML, Nascimento MIM (2015). A pesquisa histórica e a história da educação. Rev HISTEDBR On-Line.

[13] Valadares AFM, Magro MCS (2014). Opinion of nursing students on realistic simulation and the curriculum
internship in hospital setting. Acta Paul Enferm.

[14] Lancaster RJ (2014). Serious game simulation as a teaching strategy in
pharmacology. Clin Simulation Nurs.

[15] Andrade FV, Lopes AMA (2014). Análise da construção do conhecimento sobre a abordagem interacionista
estudo de caso no ambiente virtual de aprendizagem moodle. Rev AlcanCead.

[16] Khalaila R (2014). Simulation in nursing education an evaluation of students' outcomes at
their first clinical practice combined with simulations. Nurse Educ Today.

[17] Aredes NDA, Góes FSN, Silva MAI, Gonçalves MFC, Fonseca LMM (2015). Digital object in neonatal nursing: impact on student
learning. Rev Eletr Enferm.

[18] Wilson RD, Klein JD, Hagler D (2014). Computer-based or human patient simulation-based case analysis which
works better for teaching diagnostic reasoning skills?. Nurs Educ Perspect.

[19] Bloomfield JG, While AE, Roberts JD (2008). Using a computer assisted learning for clinical skills education in
nursing integrative review. J Adv Nurs.

[20] Baptista RCN, Martins JCA, Pereira MFCR, Mazzo A (2014). Students' satisfaction with simulated clinical experiences: validation
of an assessment scale. Rev. Latino-Am. Enfermagem.

[21] Park E (2013). The development and implications of a case-based computer program to
train ethical decision-making. Nurs Ethics.

[22] Anderson JK, Page AM, Wendorf DM (2013). Avatar-assisted case studies. Nurse Educator.

[23] Mayer RE, Estrella G (2014). Benefits of emotional design in multimedia instruction. Learn Instruct.

[24] Gibson S (2009). Enhancing intergenerational communication in the classroom
Recommendations for successful teacher-student relationships. Nurs Educ Perspect.

[25] Fonseca LMM, Aredes NDA, Leite AM, Santos CB, Lima RAG, Scochi CGS (2013). Evaluation of an educational technology regarding clinical evaluation
of preterm newborns Rev. Latino-Am. Enfermagem.

[26] Batista N, Batista SH, Goldenberg P, Seiffert O, Sonzogno MC (2005). O enfoque problematizador na formação de profissionais de
saúde. Rev Saúde Pública.

